# Monocytes presenting a pro-inflammatory profile persist in patients submitted to a long-term pharmacological treatment after acute myocardial infarction

**DOI:** 10.3389/fphys.2022.1056466

**Published:** 2023-01-20

**Authors:** Daniel Carneiro de Carvalho, Francisco Antonio Helfenstein Fonseca, Maria Cristina de Oliveira Izar, Ana Luíza Pereira Assunção Silveira, Izabela Dorota Tuleta, Jônatas Bussador do Amaral, Lucas Melo Neves, André Luis Lacerda Bachi, Carolina Nunes França

**Affiliations:** ^1^ Post Graduation Program in Health Sciences, Santo Amaro University, Sao Paulo, Brazil; ^2^ Department of Medicine, Cardiology Division, Federal University of Sao Paulo, Sao Paulo, Brazil; ^3^ Department of Medicine-Cardiology, Albert Einstein College of Medicine, New York, NY, United States; ^4^ ENT Research Laboratory, Otorhinolaryngology-Head and Neck Surgery Department, Federal University of Sao Paulo, Sao Paulo, Brazil

**Keywords:** monocyte subsets, atherosclerosis, acute myocardial infarction, hipolipemiant, antiplatelet

## Abstract

**Introduction:** Although it is broadly known that monocyte recruitment is involved in atherosclerosis development and that, in accordance with the microenvironment, these cells can be modulated into three well-known subpopulations: Classical (CD14++CD16−), intermediate (CD14++CD16+), and non-classical (CD14+CD16++), the effects of treatment with different pharmacological strategies (based on lipid-lowering and antiplatelets) after acute myocardial infarction upon the monocytes modulation and the role of the chemokine receptors CCR2, CCR5 and CX3CR1 in this context, are poorly understood.

**Methods:** In this study, patients [n = 148, both men (n = 105, 71%) and women (n = 43, 29%)] submitted to treatment with a 2×2 factorial design, in which they received rosuvastatin 20 mg or simvastatin 40 mg plus ezetimibe 10 mg, as well as ticagrelor 90 mg or clopidogrel 75 mg were enrolled. Monocyte subsets were analyzed by flow cytometry at baseline (BL), and after one (1-M) and 6 months (6-M) of treatment.

**Results:** Firstly, our results showed that, regardless of the treatment received, higher percentages of classical monocytes and lower of non-classical monocytes were found at the 6-M time point than BL values, whilst the percentage of intermediate monocytes was higher in all time points assessed than the other subsets. There were reductions in the CCR2 expression by non-classical and intermediate monocytes, without differences for the classical subtype. Concerning the CCR5 expression, there were reductions in the three monocyte subtypes, whereas the CX3CR1 expression increased both in intermediate and classical monocytes, without differences for non-classical monocytes. In relation to the treatment received, a higher percentage of intermediate monocytes at the 6-M time point than the values BL was observed in the group treated with simvastatin + ezetimibe + clopidogrel. No significant differences were found concerning non-classical, intermediate, and classical monocytes, for CCR2, CCR5, and CX3CR1 in the four treatment arms.

**Conclusion:** Taken together, our results demonstrated that even under lipid-lowering and antiplatelet therapy for 6 months, the inflammatory phenotype of monocytes still persisted in the patients enrolled in this study.

## 1 Introduction

Atherosclerosis is recognized as chronic inflammatory and autoimmune disease, related to cellular and humoral responses from innate and adaptive immunity (Wolf et al., 2020). In terms of innate immunity, it is well-known that monocytes/macrophages and dendritic cells are pivotal roles, in the development of atherosclerotic plaque, both in the early and advanced phases. In fact, it has been reported that, in accordance with their phenotypes, these cells can show both beneficial and harmful functions in atherosclerosis and acute myocardial infarction (AMI) (Vallejo et al., 2021).

In an interesting way, it was presented that cardiovascular risk factors and the stage of the disease can influence the phenotype of the monocyte subpopulations, which are classified into classical (CD14++CD16−), intermediate (CD14++CD16+), and non-classical (CD14+CD16++) (Patterson et al., 2022). Based on the literature, monocytes have remarkable plasticity and can modulate their phenotype according to the environment (Peet et al., 2020; Trzebanski and Jung, 2020).

Classical monocytes are described in many studies as inflammatory cells, which are able to produce many inflammatory cytokines, such as interleukins IL-1, IL-12, and tumor necrosis factor-alpha (TNF-α), and present a high expression of the monocyte chemokine receptor CCR2 (C-C chemokine receptor type 2) (Gautier et al., 2009; Wang et al., 2021). These cells comprise the majority of monocytes in humans (over 90%) and are recruited to sites of active inflammation from bone marrow and spleen (Gautier et al., 2009; Swirski, 2011; França et al., 2017; Wang et al., 2021).

On the other hand, non-classical monocytes comprise around 10% of the circulating monocytes that, in a general way, produce high levels of anti-inflammatory factors, do not express CCR2, and are recognized as patrolling cells due their prominent capacity to remove debris from the vasculature (Thomas et al., 2015; Hehenkamp et al., 2021; Tahir and Steffens, 2021).

The most recent monocyte subtypes described was intermediate, which corresponds to up to 5% of the circulating monocytes and expresses the chemokine receptors CCR2, CCR5 (C-C chemokine receptor type 5), and CX3CR1 (CX3C motif chemokine receptor 1) (Ziegler-Heitbrock et al., 2010; Kishimoto et al., 2020; Ozanska et al., 2020). These cells have a highly phagocytic capacity of removing apoptotic cells after an AMI (Zawada et al., 2011), as well as the capacity to produce high levels of reactive oxygen species and both pro- (such as TNFα and IL-1β) and anti-inflammatory cytokines (such as IL-10) after stimulation *in vitro* with LPS (Cros et al., 2010; Kishimoto et al., 2020).

It has been reported that the controlling immune and inflammatory responses could contribute not only to less infiltration of plaques by monocytes, especially by classical monocytes, but also to better ventricular remodeling (Coste et al., 2020; Casarotti et al., 2021), even though the role of subsets of monocytes after acute coronary syndromes is still poorly understood. Therefore, pharmacological strategies including the choice of lipid-lowering and antiplatelet therapy may influence monocyte differentiation and the recovery of myocardial ischemia.

In this respect, statins are lipid-lowering drugs recommended for all patients at very-high cardiovascular risk, such as subjects after AMI (Grundy et al., 2018). These drugs also exhibit pleiotropic effects that include anti-inflammatory actions (Mollazadeh et al., 2021), and improvement in immune responses (Moreira et al., 2014). Beyond the statins, antiplatelet drugs are also recommended for patients with acute coronary syndromes since may decrease the release of several pro-inflammatory cytokines (Zheng et al., 2019).

Despite of the fact that the therapy with lipid-lowering and antiplatelet drugs can impact the immune and inflammatory status, until now, there are few studies that objective investigating the effect of these drugs in modulating the subsets of monocytes in the context of cardiovascular disease. Therefore, in this study, we aimed to improve the knowledge concerning the effect of different pharmacological strategies (based on lipid-lowering and antiplatelets) in the modulation of monocyte subpopulations (classical, intermediate, and non-classical) in patients after AMI.

## 2 Materials and methods

### 2.1 Study design

This study was a prospective, randomized, open label study, with blinded analyses of endpoints (PROBE), registered as a clinical trial prior to initiation (ClinicalTrials.gov Identifier: NCT02428374) and followed the international standards of good clinical practice and data harmonization (GCP/ICH).

Patients treated by pharmacological thrombolysis in the first 6 h of ST segment elevation myocardial infarction (STEMI) were referred to Hospital Sao Paulo, Brazil for coronary angiography in the first 24 h. Before the invasive procedure, they were randomized using a central computerized system (battle-ami.huhsp.org.br) in a 2x2 factorial design to be treated with rosuvastatin 20 mg or simvastatin 40 mg plus ezetimibe 10 mg, as well as ticagrelor 90 mg or clopidogrel 75 mg, in addition to routine therapy for AMI (Coste et al., 2020). The assigned treatment was maintained for 6 months.

### 2.2 Inclusion and exclusion criteria

We included patients of both sexes under 75 years of age, with their first AMI. Patients with known intolerance to the study drugs, or comorbidities that could affect the evaluation of treatments, such as active liver disease, recent bleeding, neoplasms, cardiogenic shock, or a personal history that could prevent adequate evaluation of treatments (alcoholism, drug addiction, infectious or chronic rheumatologic diseases such as AIDS, rheumatoid arthritis, Systemic Lupus Erythematosus), were excluded.

### 2.3 Clinical and laboratory assessments

Subjects were submitted to clinical evaluations that included demographic characteristics, risk factors, measurements of body weight, height, with calculation of body mass index, assessment of systolic and diastolic blood pressure, and heart rate, according to the Update of the Brazilian Guideline on Dyslipidemia and Prevention of Atherosclerosis, 2017 (Faludi et al., 2017). Lipid profile was assayed by standard methods, blood glucose by fluorometric assay using commercial kits and automated system and glycated hemoglobin by high performance liquid chromatography. High-sensitivity troponin was measured by electrochemiluminescence.

### 2.4 Obtention of peripheral blood mononuclear cells

Approximately 10 mL of peripheral blood were collected from patients in tubes containing anticoagulant EDTA at three different times: 24 h after hospitalization (baseline—BL), and after one (1-M) and six (6-M) months of pharmacological treatment. Blood samples were initially mixed with saline (1:1 ratio), then, it was added to a tube containing Ficoll-Hypaque (Ficoll Paque Plus, GE Healthcare Bio-Sciences AB, Uppsala, Sweden) and centrifuged at 800 g, 22°C for 20 min for isolation of peripheral blood mononuclear cells by a concentration gradient. Subsequently, the cells were washed in isotonic solution (PBS). Cell viability and count were performed by using a Neubauer chamber, after staining 10 uL of cells with 90 uL of 60% Trypan Blue (Sigma-Aldrich, MO, United States) for 5 min. After that, the cells (1 × 10^6^) were centrifuged, the supernatant discarded, and the pellet frozen with 1 mL of freezing solution (DMSO + fetal bovine serum) and kept in liquid nitrogen.

### 2.5 Phenotypic characterization of monocyte subsets and expression of the proteins CCR2, CCR5, and CX3CR1

**FIGURE 1:**
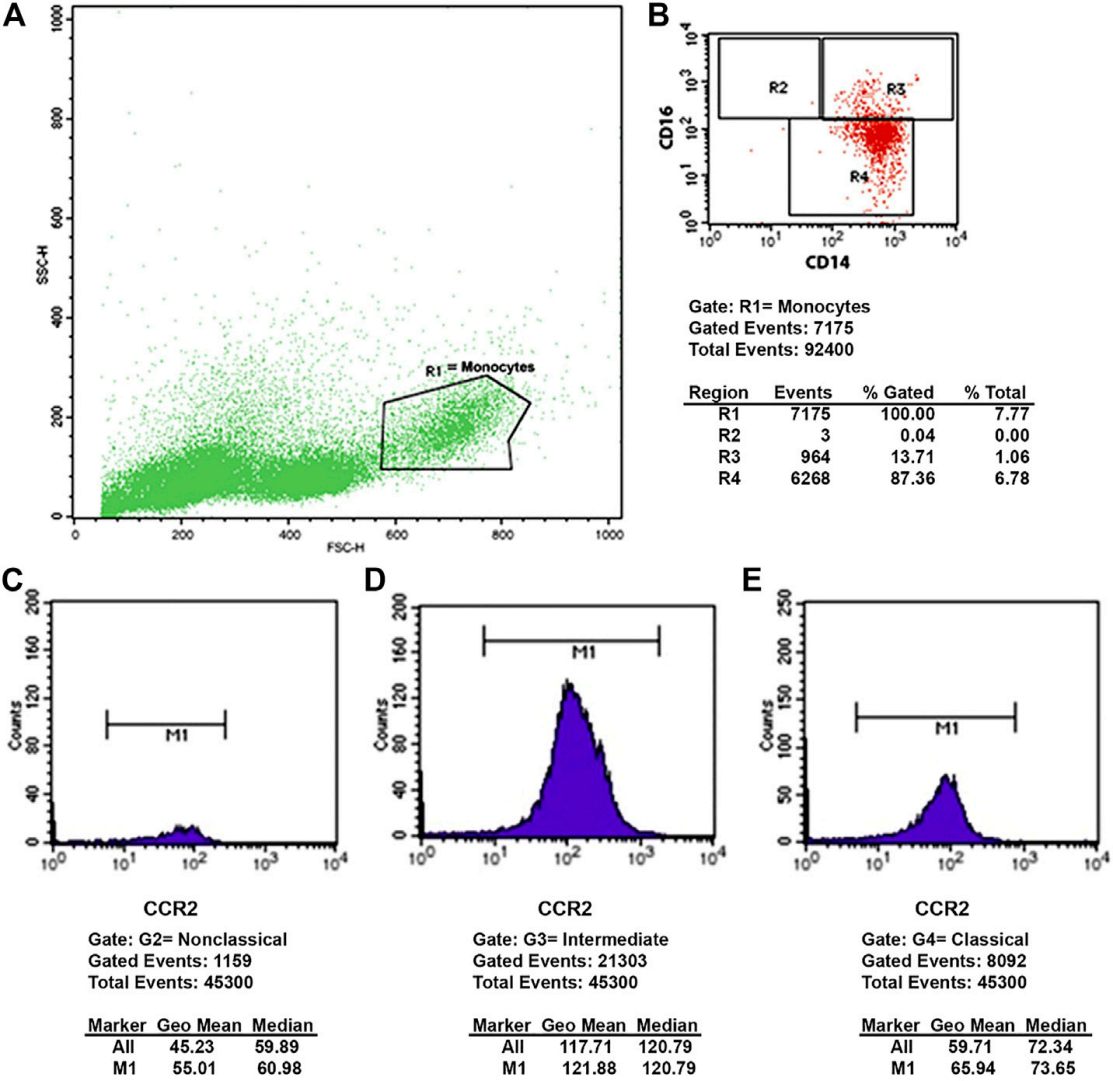
Flow cytometry plot. **(A)** Size (FSC-H) and complexity (SSC-H) graph, showing the mononuclear cells and the monocyte gate (R1). **(B)** Fluorescence graph, showing: R2 non-classical (CD14+CD16++), R3 intermediate (CD14++CD16+) and R4 classical (CD14++CD16^−^) monocyte subtypes. **(C–E)** Histograms representing the expression of CCR2 in non-classical **(C)**, intermediate **(D)** and classical **(E)** monocytes.

Peripheral blood mononuclear cells were washed, centrifuged, and immunolabeled for 30 min at 4 °C with the following antibodies: CD14 conjugated with allophycocyanin—APC or fluorescein isothiocyanate—FITC (BD Biosciences, Franklin Lakes, NJ, United States), CD16 conjugated with FITC or phycoerythrin—PE (BD Biosciences, Franklin Lakes, NJ, United States), CCR2 conjugated to Alexa Fluor^®^ 647 (BD Biosciences, Franklin Lakes, NJ, United States), CCR5 conjugated to PE (BD Biosciences, Franklin Lakes, NJ, United States) and PE-conjugated CX3CR1 (BD Biosciences, Franklin Lakes, NJ, United States). Then, the reading was immediately performed in a flow cytometer (FACSCalibur—BD Biosciences, San Jose, United States) with analysis performed by the Cell Quest Pro software, at baseline, 1 month, and after 6 months of treatment. At least 10,000 events were acquired. The expression of the receptors CCR2, CCR5, and CX3CR1 was evaluated in the three subpopulations of monocytes (classical, intermediate, and non-classical) at BL, 1-M and 6-M. One example of the CCR2 analysis is shown in Figure 1, the same was done to CCR5 and CX3CR1.

### 2.6 Statistical analysis

For data analysis, SPSS version 18.0 was used. Descriptive data are presented as percentages, mean ± standard error (SE) or median with interquartile range (IQR), when appropriate. Comparisons between the three time-points (baseline, one and 6 months) for all participants, regardless of the treatment, were obtained by the ANOVA test, Bonferroni *post hoc* test (lipid profile) and Friedman test (monocyte subtypes). The comparisons between monocyte subtypes in the four treatment arms were performed by the Kruskal-Wallis test. The significance level was set at *p* <.05.

### 2.7 Ethical aspects

The study was approved by the Research Ethics Committee of the Universidade Santo Amaro—UNISA (under number 2.275.621) and followed the principles described in the Helsinki Declaration.

## 3 Results

Table 1 presents the main characteristics of the study population [n = 148, both men (n = 105, 71%) and women (n = 43, 29%)] separated into the respective therapeutical arms, as well as parameters of kidney function, diabetes, and myocardial injury assessed in the first 24 h of hospitalization.

**TABLE 1:** General characteristics from the participants of the study.

	RT (n = 43)	RC (n = 37)	SET (n = 35)	SEC (n = 33)
Age, median (IQR), years	58 (51–64)	59 (53–64)	58 (53–64)	59 (53–67)
Male sex,n (%)	29 (67)	26 (70)	25 (71)	25 (76)
Weight, mean (SE), Kg	78.34 (2.31)	73.17 (2.29)	76.39 (3.36)	73.30 (2.46)
Height, mean (SE), m	1.69 (.01)	1.65 (.01)	1.64 (.02)	1.63 (.02)
Body mass index, mean (SE), kg/m^2^	27.25 (.74)	27.21 (.71)	28.33 (1.11)	27.29 (1.05)
Systolic blood pressure, mean (SE), mmHg	123 (3)	126 (4)	129 (4)	124 (4)
Diastolic blood pressure, mean (SE), mmHg	76 (2)	77 (2)	78 (2)	75 (3)
Diabetes*, n (%)	32 (74)	22 (81)	22 (63)	19 (58)
Hypertension, n (%)	32 (74)	26 (71)	26 (75)	23 (70)
Tabagism, n (%)	32 (74)	29 (79)	26 (75)	25 (77)
HbA1c, mean (SE), (%)	7.2 (.3)	6.2 (.2)	6.2 (.2)	6.3 (.3)
Glucose, mean (SE), mg/dL	154 (11)	137 (9)	136 (11)	136 (11)
Troponin, mean (SE), picomol/L	8,542 (2,346)	7,740 (838)	7,460 (1172)	7,092 (1099)
Creatinine, mean (SE), mg/dL	.91 (.03)	.93 (.06)	1.00 (.06)	1.00 (.06)
GFR, mL/min/1.73m^2^	84 (3)	88 (3)	80 (3)	82 (4)
WBC baseline, mean (SE), mm^3^	11,843 (3,827)	13,265 (4,364)	12,224 (4,874)	12,522 (4452)
Lymphocyte baseline, mean (SE), mm^3^	2,228 (859)	2,343 (1,039)	2,369 (1,099)	1845 (589)
Non-Lymphocyte baseline, mean (SE), mm^3^	5,31 (4,27)	5,69 (3,52)	5,27 (3,99)	7,62 (916)
LVEF 1 month, mean (SE), %	48 (12)	47 (12)	48 (12)	49 (11)

Values express the means and standard error (SE), or median (quartile range) or percentagem for categorical variables; RT, Rosuvastatin + Ticagrelor; RC, Rosuvastatin + Clopidogrel; SET, Simvastatin + Ezetimibe + Ticagrelor; SET, Simvastatin + Ezetimibe + Clopidogrel; *Non-diabetic, HbA1c < 5.7%; pre-diabetic, HbA1c between 5.7% and 6.4%; diabetic ≥6.5%; GFR, Estimated glomerular filtration rate (CKD-EPI); LVEF, left ventricular ejection fraction.

The lipid profile of patients throughout the study is shown in Table 2. Comparisons between baseline (BL), 1 month (1-M) and 6 months (6-M) after AMI showed reductions in total cholesterol, LDL-C, and non-HDL-C after 1-M and 6-M as compared to the BL values, as well as an increase in HDL-C after 6-M as compared to BL (or 1-M) values, with no differences for triglycerides.

**TABLE 2:** Lipid profile during the study.

Variables	Mean (SE) baseline	Mean (SE) one month	Mean (SE) six months	p*
CT	202 (4)	126 (3)	127 (3)	<.0001
LDL-C	131 (3)	65 (2)	64 (2)	<.0001
HDL-C	41 (1)	38 (1)	45 (1)	.001
Non-HDL-C	121 (6)	87 (3)	87 (3)	<.0001
Triglycerides	175 (14)	152 (12)	140 (7)	.098

Values represent means and standard errors (SE), mg/dL. CT, total cholesterol; LDL-C, cholesterol from low density lipoprotein; Non-HDL-C, non-HDL-C; HDL-C, cholesterol from high density lipoprotein. *ANOVA, test, Bonferroni *post hoc* test.

Table 3 presents the percentages obtained for each monocyte subtype for all participants of the study, regardless of the treatment arm. It was observed a reduction in the non-classical monocytes (*p* <.0001) after 6 months (6-M) as compared to the baseline (BL) values, whereas the percentage of classical monocytes was higher (*p* <.0001), with no differences for intermediate monocytes (*p* = .299). Furthermore, by the Kruskal-Wallis test, it was found that the percentage of intermediate monocytes was higher in all time points than the values observed to other subsets (*p* <.0001).

**TABLE 3:** Percentage of monocyte subtypes, throughout the study.

Monocytes	Baseline	One month	Six months	p intra groups	p between groups		
					Baseline	One month	Six months
Classical	28.89 (1.79)	28.00 (1.62)	36.09 (1.97)	<.0001			
Intermediate	51.04 (2.22)	57.86 (1.94)	55.40 (1.99)	.299	<.0001	<.0001	<.0001
Non-classical	5.23 (.96)	6.53 (1.07)	1.30 (.33)	<.0001			

Data represent means (standard errors of means–SEM). Friedman test, intra group comparisons (baseline × 1 month × 6 months) and Kruskal-Wallis test, comparisons between groups (classical × intermediate × non-classical).

Tables 4–6 represent the expression of proteins (CCR2, CCR5, and CX3CR1) in monocyte subtypes (non-classical, intermediate, and classical subtypes) by flow cytometry, at the three visits, regardless of the treatment arm. Compared to the baseline (BL) values, as shown in Table 4, there were reductions in the CCR2 expression in non-classical and intermediate monocytes, without differences for the classical subtype (*p* = .009; *p* = .011 and *p* = .175, respectively. Friedman test). In addition, the intermediate monocytes showed higher expression of CCR2 than the other subtypes, at baseline, one and 6 months (*p* < .0001 for the three cases. Kruskal-Wallis test).

**TABLE 4:** Level of expression (a.u.) of the receptor CCR2 in monocyte subtypes, throughout the study.

Monocytes	Baseline	One month	Six months	p intra groups*	p between groups#		
					Baseline	One month	Six months
Classical	90.98 (10.72)	180.62 (93.76)	76.32 (4.29)	.175			
Intermediate	228.72 (38.47)	209.98 (23.78)	161.08 (10.68)	.011	<.0001	<.0001	<.0001
Non-classical	65.80 (4.63)	66.38 (3.94)	57.55 (3.22)	.009			

Data represent means (standard errors of means–SEM). *Friedman test, intra group comparisons (baseline × 1 month × 6 months) and #Kruskal-Wallis test, comparisons between groups (classical × intermediate × non-classical);.au, fluorescence arbitrary units.

Concerning the CCR5 expression (Table 5), there were higher expression for classical and intermediate monocytes and reduction in non-classical (*p* <.0001, *p* <.0001, and *p* = .003, respectively. Friedman test). The intermediate monocytes had higher expression of CCR5 at baseline and after six-months of treatment (*p* <.0001 for both cases. Kruskal-Wallis test) and classical monocytes showed higher expression of CCR5 after 1 month of treatment (*p* <.0001 Kruskal-Wallis test).

**TABLE 5:** Level of expression (a.u.) of the receptor CCR5 in monocyte subtypes, throughout the study.

Monocytes	Baseline	One month	Six months	p intra groups*	p between groups#		
					Baseline	One month	Six months
Classical	5.23 (.30)	5.29 (.16)	5.81 (1.04)	<.0001			
Intermediate	6.61 (1.51)	4.12 (.23)	6.96 (2.15)	.003	<.0001	<.0001	<.0001
Non-classical	4.95 (1.24)	3.31 (.12)	3.99 (.59)	<.0001			

Data represent means (standard errors of means–SEM). *Friedman test, intra group comparisons (baseline × 1 month × 6 months) and #Kruskal-Wallis test, comparisons between groups (classical × intermediate × non-classical); au, fluorescence arbitrary units.

Besides, the CX3CR1 expression increased both in classical and intermediate monocytes, without differences for non-classical monocytes (Table 6, *p* <.0001, *p* = .049, and *p* = .138, respectively. Friedman test). The intermediate monocytes had higher expression of CX3CR1 at baseline, one and 6 months of treatment (*p* <.0001 in the three cases. Kruskal-Wallis test).

**TABLE 6:** Level of expression (a.u.) of the receptor CX3CR1 in monocyte subtypes, throughout the study.

Monocytes	Baseline	One month	Six months	p intra groups*	p between groups#		
					Baseline	One month	Six month
Classical	81.70 (5.75)	92.01 (7.82)	100.64 (5.56)	<.0001			
Intermediate	273.38 (18.27)	300.78 (22.94)	324.45 (30.89)	.049	<.0001	<.0001	<.0001
Non-classical	62.17 (5.21)	86.44 (15.66)	171.69 (61.43)	.138			

Data represent means (standard errors of means–SEM). *Friedman test, intra group comparisons (baseline × 1 month × 6 months) and #Kruskal-Wallis test, comparisons between groups (classical × intermediate × non-classical); au.: fluorescence arbitrary units.

The percentages of the monocyte subsets in the four groups, based on the therapeutical treatments, are shown in Table 7. It was found a higher percentage of intermediate monocytes in the group treated with simvastatin + ezetimibe + clopidogrel group (*p* = .020). In addition, the percentage of intermediate monocytes was higher than the other subsets in the four arms of treatment (*p* <.0001 for all groups. Kruskal-Wallis test).

**TABLE 7:** Percentage of monocyte subtypes, according to the treatment arms.

Monocytes	RT	RC	SET	SEC	p between groups*	p between groups #			
						RT	RC	SET	SEC
Classical	30.73 (1.77)	31.48 (2.17)	34.10 (2.41)	27.44 (2.11)	.158				
Intermediate	53.11 (2.27)	52.49 (2.50)	51.45 (2.60)	61.65 (2.11)	.020	<.0001	<.0001	<.0001	<.0001
Non-classical	3.90 (.90)	4.84 (1.21)	4.41 (1.02)	4.52 (.97)	.097				

Data represent means (standard errors of means–SEM). Kruskal-Wallis test, *comparisons between monocyte groups (classical × intermediate × non-classical) and #comparisons between treatment arms. RT, Rosuvastatin + Ticagrelor; RC, Rosuvastatin + Clopidogrel; SET, Simvastatin + Ezetimibe + Ticagrelor; SEC, Simvastatin + Ezetimibe + Clopidogrel.

Tables 8–10 show the comparison between the four treatment arms, concerning the expression of CCR2, CCR5, and CX3CR1. No significant differences were found concerning non-classical, intermediate, and classical monocytes, for CCR2 (Table 8, *p* = .890; *p* = .731 and *p* = .824), CCR5 (Table 9, *p* = .658; *p* = .639 and *p* = .458) and CX3CR1 (Table 10, *p* = .419; *p* = .124 and *p* = .127), respectively (Kruskal-Wallis test).

**TABLE 8:** Level of expression (a.u.) of the receptor CCR2 in monocyte subtypes, according to the treatment arms.

Monocytes	RT	RC	SET	SEC	p between groups*	p between groups #			
						RT	RC	SET	SEC
Classical	87.01 (7.42)	214.78 (123.46)	86.03 (6.36)	74.43 (4.09)	.705				
Intermediate	188.28 (17.51)	204.49 (27.17)	210.23 (24.08)	201.46 (47.93)	.670	<.0001	<.0001	<.0001	<.0001
Non-classical	63.16 (3.83)	64.28 (5.08)	65.70 (4.94)	60.61 (4.71)	.863				

Data represent means (standard errors of means–SEM). Kruskal-Wallis test, *comparisons between treatment arms and # comparisons between groups of monocytes (classical × intermediate × non-classical). RT, Rosuvastatin + Ticagrelor; RC, Rosuvastatin + Clopidogrel; SET, Simvastatin + Ezetimibe + Ticagrelor; SEC, Simvastatin + Ezetimibe + Clopidogrel; au, fluorescence arbitrary units.

**TABLE 9:** Level of expression (a.u.) of the receptor CCR5 in monocyte subtypes, according to the treatment arms.

Monocytes	RT	RC	SET	SEC	p between groups*	p between groups #			
						RT	RC	SET	SEC
Classical	5.29 (.24)	6.30 (1.35)	4.96 (.38)	5.18 (.22)	.263				
Intermediate	5.10 (.59)	9.21 (3.27)	4.69 (.71)	4.54 (.87)	.424	.002	.019	.012	.030
Non-classical	3.63 (.23)	5.71 (1.79)	3.53 (.36)	3.47 (.34)	.302				

Data represent means (standard errors of means–SEM). Kruskal-Wallis test, *comparisons between treatment arms and # comparisons between groups of monocytes (classical × intermediate × non-classical). RT: Rosuvastatin + Ticagrelor; RC: Rosuvastatin + Clopidogrel; SET: Simvastatin + Ezetimibe + Ticagrelor; SEC: Simvastatin + Ezetimibe + Clopidogrel;.au, fluorescence arbitrary units.

**TABLE 10:** Level of expression (a.u.) of the receptor CX3CR1 in monocyte subtypes, according to the treatment arms.

Monocytes	RT	RC	SET	SEC	p between groups*	p between groups #			
						RT	RC	SET	SEC
Classical	95.25 (9.80)	90.86 (5.47)	97.89 (6.48)	81.82 (6.43)	.126				
Intermediate	268.87 (23.04)	313.81 (29.95)	360.67 (37.75)	264.54 (21.93)	.086	<.0001	<.0001	<.0001	<.0001
Non-classical	63.13 (6.31)	119.54 (40.41)	193.20 (81.16)	64.09 (5.90)	.334				

Data represent means (standard errors of means–SEM). Kruskal-Wallis test, *comparisons between treatment arms and # comparisons between groups of monocytes (classical × intermediate × non-classical). RT, Rosuvastatin + Ticagrelor; RC, Rosuvastatin + Clopidogrel; SET, Simvastatin + Ezetimibe + Ticagrelor; SEC, Simvastatin + Ezetimibe + Clopidogrel; a.u, fluorescence arbitrary units.

## 4 Discussion

To the best of our knowledge, no follow-up studies have evaluated the chronic effect (6-M) of pharmacological therapies, involving the lipid-lowering rosuvastatin, and simvastatin + ezetimibe, plus the antiplatelet agents ticagrelor and clopidogrel, on monocyte subsets and expression of the chemokine receptors CCR2, CCR5, and CX3CR1 after AMI in humans. The cross-sectional studies published showed comparisons between patients after AMI and healthy individuals, evaluating only the acute phase. Studies evaluating chronic effects on monocyte subsets are scarce.

Our main findings showed higher percentages of classical monocytes and lower of non-classical monocytes after 6 months of treatment, regardless of the choice of the pharmacological strategy. Concerning monocyte subsets, it has been reported that classical monocytes show a prominent proinflammatory function (Gautier et al., 2009), whereas non-classical monocytes, on the other hand, present a more anti-inflammatory phenotype and the remarkable ability to remove debris from the vasculature (patrolling) (Thomas et al., 2015).

Based on the data presented by Berg et al. (2012), which compared a group of individuals with at least one outcome during a 15 years follow-up with a control group without outcomes, higher levels of classical monocytes were evidenced in the case group. Furthermore, the same authors also reported the lowest event-free survival among participants with the highest level of these cells, suggesting that classical monocytes predict cardiovascular events. Corroborating these informations,Höpfner et al. (2019) found an elevation of classical monocytes in hospitalized patients with coronary heart disease, without correlations between intermediate and non-classical monocytes with cardiac outcomes, suggesting that the classical subtype is predictive for major cardiac events.

Beyond these findings, Zeynalova et al. (2021) evaluated the monocytes subsets in patients with different cardiovascular risks, without previous AMI, according to the Framingham Risk Score (FRS), and it was verified not only lower monocyte counts of the three subsets in patients with lower cardiovascular risks as well as a continuous increase of these cells in the higher risk patients. Moreover, Leers et al. (2017) evaluated the monocyte subsets in acute coronary syndrome (ACS) spectrum [unstable angina pectoris (UAP), non-ST-segment elevation myocardial infarction (NSTEMI), and ST-elevation myocardial infarction (STEMI)]. These authors found higher levels of the three monocyte subsets in patients with AMI (NSTEMI or STEMI) when compared to patients without ACS or with UAP, indicating an increase in these leukocytes related to the severity of the ACS.

Marsh et al. (2021) evaluated circulating monocytes during acute STEMI, from immediately prior to a primary percutaneous coronary intervention to 90 min post-reperfusion. The authors reported a remarkable reduction in the three monocyte subsets, mainly non-classical monocytes, which was directly correlated with larger infarct size and impaired LVEF (Left ventricular ejection fraction). According to the same authors, some studies evidence a more reparative role of non-classical monocytes in the AMI context, their results suggest an aggravation of local inflammation in the earlier AMI promoted by non-classical monocytes.

Particularly in this study, we evidenced a higher percentage of intermediate monocytes, when compared to the other subpopulations, in the three time-points (24 h after AMI, and after one and 6 months of treatment). Few studies describe the true role of this monocyte subtype in the atherosclerosis/AMI context; however, some studies indicate that this subtype presents a high phagocytic capacity, which favors the removal of apoptotic cells after AMI (Zawada et al., 2011), and represent the most inflammatory of the subtypes of monocytes (Almubarak et al., 2020).

In accordance with the study of Lu et al. (2015), in which it was investigated the role of intermediate monocytes in post-AMI patients, these cells are closely associated with the extent of infarction and can also be useful to predict new cardiovascular events. In this sense, Rogacev et al. (2012) analyzed intermediate monocytes in 951 individuals eligible for coronary angiography and showed an association between this subtype of monocytes and cardiovascular events, during a follow-up period of 2.6 years. In another study from the same group, in which it was evaluated the relationship between monocyte subsets and cardiovascular events in patients with chronic kidney disease, the authors showed that intermediate monocytes are independently associated with future cardiovascular events in the analyzed patients (Ozanska et al., 2020). Taken together, these data corroborate our findings, indicating an important relationship between intermediate monocytes and cardiovascular complications.

Another important finding presented in this study was related to the fact that although submitted to high-intensity lipid-lowering and antiplatelet treatments for AMI, patients still presented a residual inflammatory risk after 6 months of the treatment since it was found higher percentages of pro-inflammatory monocyte subtype. In agreement with the literature, this residual inflammatory risk could be putatively explained by the trained immunity, which refers to a persistent pro-inflammatory phenotype (for months to ≤1 year) (Kleinnijenhuis et al., 2014) after exposure to atherogenic compounds, such as oxidized low-density lipoprotein (oxLDL) (Bekkering et al., 2014). Epigenetic reprogramming of histone modifications, as well as metabolic changes, which can occur from the myeloid progenitors in the bone marrow (Christ et al., 2018; Mitroulis et al., 2018), are responsible for driving to this trained immunity (Netea et al., 2016).

Interestingly, Bekkering et al. (2019) evaluated the trained immunity in patients with familial hypercholesterolemia, under treatment for 3 months with statins, and, even though the authors did not observe differences in the pro-inflammatory monocyte counts after treatment, there was a reduction in the cholesterol levels. Furthermore, the same authors also compared patients with severe symptomatic coronary atherosclerosis and patients with mild asymptomatic atherosclerosis, and it was observed persistent hyperresponsiveness in the monocytes of the patient group with severe symptoms since these cells maintained the production of proinflammatory cytokines, which was not found in the patient group with mild symptoms (Bekkering et al., 2016).

Particularly, we also investigate the effect of four pharmacological strategies, based on lipid-lowering and antiplatelets, on the percentages of monocyte subsets. We were able to demonstrate that, only in the simvastatin + ezetimibe + clopidogrel group, the percentages of intermediate monocytes were increased.

It is utmost of to point out that it was expected a less potent anti-inflammatory action for this treatment arm, since clopidogrel has metabolism by cytochrome P450 CYP 3A4, the same site of metabolism as simvastatin (but not rosuvastatin), leading to a decrease of the magnitude of the pleiotropic effect anti-inflammatory promoted by simvastatin. In addition, simvastatin not only presents a shorter half-life than rosuvastatin but also less potent inhibition in cholesterol synthesis. These pieces of information can help us to understand our findings, due to the anti-inflammatory effects mediated by statins are dependent on the lower activation of the endogenous pathway of cholesterol synthesis (Cai et al., 2021).

In a different way of our data, the results presented by Belhassena et al. (2020) in a study in which the authors evaluated the effects of aspirin (acetylsalicylic acid—ASA) on immunomodulation of monocytes from patients with AMI and controls, the cells treated *ex vivo* with different concentrations of ASA showed a phenotypic modulation of the monocytes to a lower expression of CD16, and considered these cells as an anti-inflammatory profile. These apparently contradictory findings might be related to the different methodological aspects: 1) antiplatelet drugs utilized (in our study we used clopidogrel and ticagrelor); 2) the monocytes were treated *ex vivo* for few hours in the Belhassena study; and, 3) few patients were analyzed by Belhassena.

In the current study, we suggest a persistence of the inflammatory phenotype after 6 months of treatment, despite the highly-effective lipid-lowering and antiplatelet therapies, due to an increase in classical (proinflammatory) monocytes and a reduction in non-classical (antiinflammatory) monocytes. This might be related to trained immunity, defined as a persistence of a proinflammatory phenotype, regardless of the choice of the pharmacological strategy. Furthermore, the observation of high levels of intermediate monocytes after 6 months of treatment can reinforce the role of these cells with cardiovascular complications.

Beyond these findings, the expression of proteins CCR2, CCR5, and CX3CR1 in the cell membrane of monocyte subsets was also evaluated by flow cytometry, at the three visits. Although no significant differences were found in the analysis involving the treatment arms applied, the analysis of the results obtained regardless of the treatment imposed showed significant results. One interesting finding was the higher expression of the receptors CCR2, CCR5 and CX3CR1 in intermediate monocytes compared to the other subtypes, even at the end of the treatment, which could reinforce the previous suggestion that a trained immunity may be involved in the study context, since the intermediate monocytes are described in the literature as the most inflammatory of the three subtypes (Almubarak et al., 2020).

In addition, it was observed that, as compared to the baseline values, the CCR2 expression was significantly reduced only in the non-classical and intermediate monocytes, without differences for the classical subtype, whereas the CCR5 expression was increased in classical and intermediate monocytes and reduced in non-classical subtype, as well as the CX3CR1 expression increased both in intermediate as classical monocytes, without alterations for non-classical monocytes.

Regarding these chemokine receptors and cardiovascular diseases (CVDs), it was shown that they are crucial to promoting monocytes recruitment into the atherosclerotic plates since the disruption of the interaction between CCR2 and its ligand CCL2 (MCP-1, monocyte chemoattractant protein 1) or between CX3CR1 and its ligand CX3CL1 (fractalkine) was able to minimize the atherosclerosis progression by reducing the number of circulating monocytes (Tacke et al., 2007; Combadière et al., 2008; Saederup et al., 2008). Of interest, Berg et al. (2012) showed that even though the expression of CCR2, CCR5, and CX3CR1 was not different in the monocyte’s subsets in groups of individuals who presented CVD and controls, exclusively the CCR5 expression on non-classical monocytes (CD14+CD16++) showed a negative association with the intima-media thickness (IMT) of the carotid. Likewise, it was also reported that the individuals presenting mutation in the CCR5 gene, which leads to expressing a truncated non-functional protein, showed a decreased carotid IMT (Afzal et al., 2008). Furthermore, it had been observed that the treatment with anti-CCR5 antibody was able to inhibit the entry of CD14+CD16++ monocytes into plates in an experimental model of atherosclerosis (Weber et al., 2000; Tacke et al., 2007). Based on these pieces of information, our findings of a significant reduction of CCR5 expression in non-classical (reparative) monocytes after AMI are in accordance to the Berg’s results and reinforce the role of the monocytes expressing CCR5 in the CVD context.

Concerning CCR2 expression, there were reductions in intermediate and non-classical monocytes, without differences in the classical monocytes after the treatment. As formerly reported, the classical monocytes not only are the main circulating monocytes but also express high levels of CCR2 (Gautier et al., 2009; Wang et al., 2021). In terms of CVDs, it has been reported that, both in mice and humans, elevations in CCR2 expression are closely associated with atherosclerosis progression and CVDs since it was observed that monocytes CCR2-are not able to migrate through the endothelium, in response to CCL2 (MCP-1), due to its less capacity of adhering to these vascular cells (Han et al., 1999), which consequently led to reducing the number of monocytes, particularly classical monocytes, into the atherosclerotic plaque (França et al., 2017). Interestingly, Yang et al. (2015) showed that patients with unstable angina treated with atorvastatin presented decreased CCR2 expression in CD14+ monocytes after percutaneous coronary intervention (PCI) compared to the control group due to the anti-inflammatory activity of the atorvastatin, as suggested by the authors. Despite the CCR2 levels being unchanged in classical monocytes, the significant reductions found in the other monocytes subsets after AMI, regardless of the treatment arms applied, are very intriguing and demonstrate that during acute AMI phasis the levels of this receptor were increased in these cells, which is not habitual, mainly in non-classical monocytes since are recognized by do not express CCR2 (Thomas et al., 2015; Hehenkamp et al., 2021; Tahir and Steffens, 2021). Based on these observations, we can putatively suggest that the higher expression of CCR2 on the monocytes subsets during the acute AMI phasis can be a corollary factor to the atherosclerosis development since, as previously reported, the atherosclerotic plaque is more stable in CCR2 deficient mice (Schober et al., 2004) and also that higher levels of monocytes expressing CCR2 can predict cardiovascular events (Berg et al., 2012). Moreover, the significant reduction of CCR2 in intermediate and non-classical monocytes can allow us also to suggest that the monocytes’ “status” in the participants of this study returned to normal conditions.

Similarly to CCR5, the levels of CX3CR1 protein were increased in the intermediate and classical monocyte subsets after AMI. Firstly, it is noteworthy to mention that the lack of alteration in the non-classical monocytes is in accordance with the literature since CX3CR1 is widely expressed in the CD16-positive monocytes (Leers et al., 2017). Secondly, it has been reported that the driving of the non-classical subset to the tissues that present inflammation is a CX3CR1-dependent process (Berg et al., 2012; Westhorpe et al., 2014). Thirdly, in agreement with the literature, CX3CR1 is also important to provide a survival signal to monocytes (Thomas et al., 2015). In an interesting way, whereas the classical monocytes are associated with a pro-inflammatory pattern, the non-classical monocytes are associated with a patrolling behavior, especially along the vasculature, which is dependent on high expression of CX3CR1 (Thomas et al., 2015). According to Marsh et al. (2017), the remarkable patrolling of this type of monocyte occurs slowly and independently of the blood flow direction. Through an experimental model of cardiac injury, particularly related to myocardial infarction, it was observed that monocyte depletion not only significantly decreased ventricular function as well as raised mortality (van Amerongen et al., 2007). Thus, these data can reinforce the pivotal action of monocytes both in the wound healing and repair of heart tissue after cardiac injury (Liu et al., 2010). Moreover, it was reported that patients with restenosis presented a higher count of CD14+CD16+CX3CR1+ monocytes than patients without restenosis after 9 months of the implantation of the bare-metal stent as a result of acute myocardial infarction (Liu et al., 2010). Taken these pieces of information into account, our findings that the levels of CX3CR1 protein increased after AMI, regardless of the treatment imposed, allows us to putatively suggest that not only the survival but also the patrolling monocytes-related could be improved. However, we agreed that the consequences of these observations along the time needs to be better understand in future studies.

## Data Availability

The raw data supporting the conclusion of this article will be made available by the authors, without undue reservation.
